# Acupuncture for dry eye syndrome after refractive surgery: study protocol for a randomized controlled trial

**DOI:** 10.1186/1745-6215-14-351

**Published:** 2013-10-24

**Authors:** Hyesun Jang, Sanghun Lee, Tae-Hun Kim, Ae-Ran Kim, Minhee Lee, Jun-Hwan Lee

**Affiliations:** 1 Acupuncture, Moxibustion and Meridian Research Group, Medical Research Division, Korea Institute of Oriental Medicine, 1672 Yuseongdaero, Yuseong-gu 305-811, Daejeon, South Korea; 2 College of Korean Medicine, Gachon University, Seongnam, South Korea

**Keywords:** Acupuncture, Dry eye, Refractive surgery

## Abstract

**Background:**

Dry eye syndrome is a frequent complication of refractive surgery. Acupuncture has been widely used to alleviate the associated symptoms. However, the use of acupuncture for patients who suffer from dry eye syndrome following refractive surgery has certain drawbacks. This pilot study aims to evaluate the efficacy of acupuncture treatment in treating the signs and symptoms of dry eye syndrome after refractive surgery.

**Methods/design:**

Forty participants will be randomly assigned to the acupuncture plus usual care group or the usual care control group. The acupuncture plus usual care group will undergo treatments on seventeen acupuncture points, three times per week for four weeks. The control group will receive only usual care during the same period. The primary outcomes will be scores on the Ocular Surface Disease Index (OSDI) and the results of examinations at 1, 3, 5, and 13 weeks. The secondary outcomes will be Tear Film Break-up Time (TBUT), as well as scores on the Schirmer-I test, visual analog scale (VAS), and quality of life (QOL) questionnaire for the self-assessment of ocular discomfort. Digital photographs will be taken to document the pattern of fluorescein staining observed on the corneal surface. The results of the Schirmer-I test, TBUT, and fluorescein-stained corneal surface digital photographs will be assessed at the screening and at week 13. VAS scores will be assessed at the screening, as well as at 1, 3, 5, and 13 weeks. QOL will be evaluated at 1, 3, 5, and 13 weeks.

**Discussion:**

This trial will provide primary data with which to investigate the clinical effectiveness and safety of acupuncture treatment for dry eye syndrome after refractive surgery.

**Trial registration:**

Current Controlled (Identifier: KCT0000727)

## Background

Refractive surgery involves the use of an excimer laser to ablate the cornea, thereby correcting the patient’s refractive error [1]. Various types of refractive surgery have been developed: photorefractive keratectomy (PRK), laser *in situ* keratomileusis (LASIK), and laser-assisted subepithelial keratomileusis (LASEK). Since the excimer laser was first used to reshape the cornea, the surgery has been performed on approximately 100,000 patients in Korea each year [2]. Various other ophthalmological advancements have improved the safety, efficacy, and predictability of surgical outcomes [3,4]. Despite these advances, however, certain limitations and complications remain. These include infection, ectasia, diffuse lamellar keratitis, subepithelial haze, dry eye, epithelial ingrowth, and the development of a button-hole flap [5].

Up to 94.8% of patients experience dry eye symptoms after refractive surgery [6]. The National Evidence-based healthcare Collaborating Agency (NECA) reported that 17% of refractive surgery patients later suffer from chronic dry eye [2].

The use of artificial tears and lifestyle modifications are currently the most common approaches to management. However, the preservatives in artificial tears may exacerbate ocular surface inflammation, and the safety of anti-inflammatory treatment is not well established [7,8]. In this context, complementary and alternative medicine can do much to alleviate the patient’s pain [9-14]. This pilot study focuses on the efficacy of acupuncture in relieving the signs and symptoms of chronic dry eye syndrome after refractive surgery.

## Methods/design

### Study design

**Figure 1:**
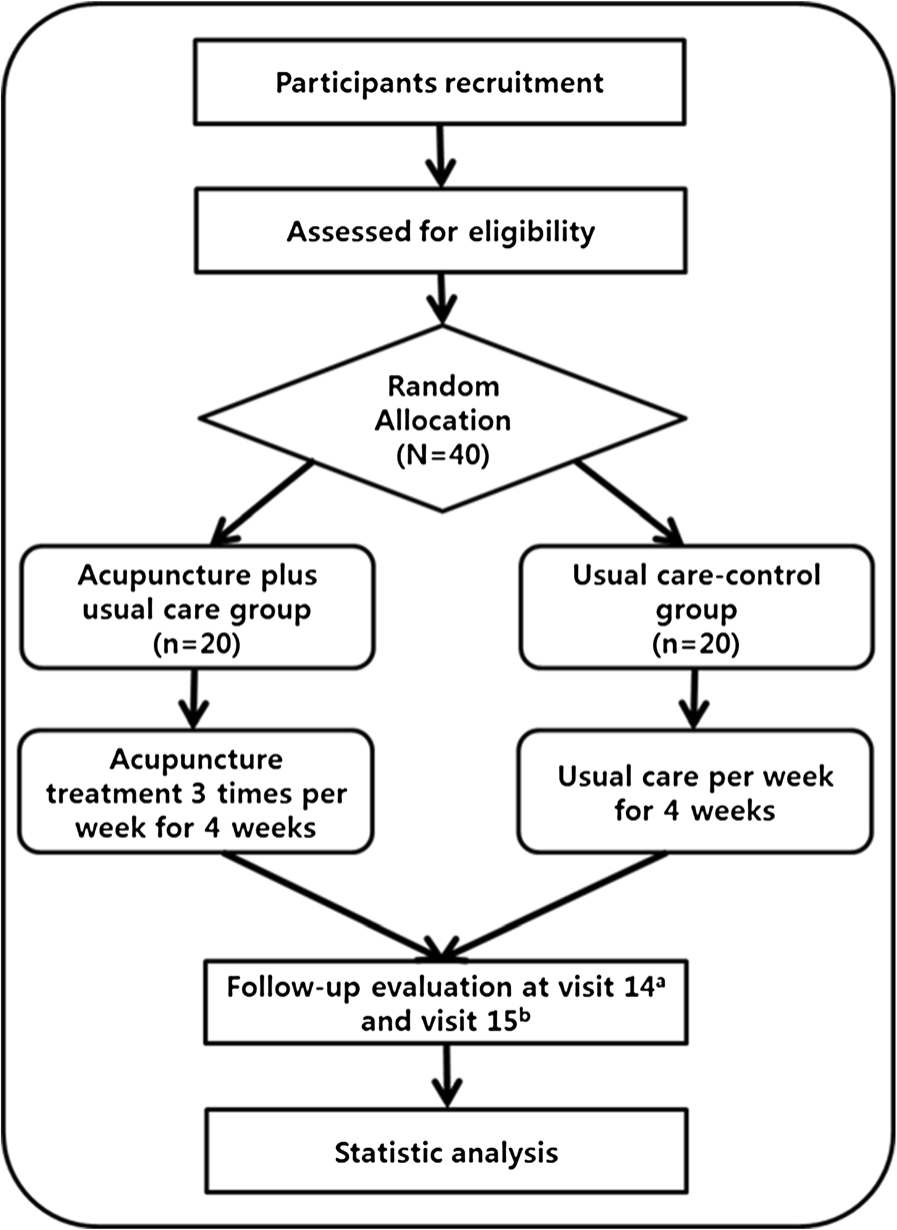
**Study flow chart.** a: 5 week (4 weeks after visit 2 + within 3 days), b: 13 week (12 weeks after visit 2 + within 3 days).

This is a randomized, controlled pilot trial. All participants will be randomly allocated to the acupuncture plus usual care group (n = 20) or the usual care control group (n = 20). The acupuncture plus usual care group will receive 12 sessions of treatment over a four-week period. The study design is depicted in Figure 1.

### Ethics

This study was approved by the Institutional Review Board (IRB) at the Oriental Hospital of Daejeon University, Korea (IRB approval number: djomc-98-1) and was registered with the Clinical Research Information Service at the Korea National Institute of Health (KCT0000727). Each participant will provide written informed consent prior to participation in the study.

### Recruitment

Participants will be recruited through advertisements in the local newspaper, distributed leaflets, the university homepage, and posts on notice boards. At the first (screening) visit, each participant’s demographic information (including ocular pathologies and current/prior use of medications and/or lubricant eye drops) will be recorded. Study participants who have experienced dry eye symptoms for at least 24 months after undergoing refractive surgery will be enrolled in the study. Patients will be included only if their ocular discomfort is rated ≥ 40 on the visual analog scale (VAS) and if the associated ophthalmological test results are abnormal (that is, Tear Film Break-up Time (TBUT) ≤ 10 seconds, ≤ 10 mm/5 minutes on the Schirmer-I test) [15] (Table 1).

**Table 1:** Inclusion and exclusion criteria

Inclusion criteria	1. Men and women, 18 to 65 years old
	2. Dry eye syndrome in one or both eyes (ICD-10:H04.1) with:
	2-1. Refractive surgery within 24 months and dry eye symptoms (for example, ocular itching, foreign body sensation, burning, pain and dryness, visual disturbance, ocular redness, and/or a sensation of tearing) of at least moderate severity
	2-2. VAS score ≥ 40
	2-3. TBUT ≤10 seconds and Schirmer-I test results ≤ 10 mm/5 minutes
	3. Voluntary participation and written informed consent
Exclusion criteria	1. Dry eye symptoms due to eyelid or eyelash defects
	2. Acute infection of the eyelid, eyeball or periorbital area
	3. Skin disease such as Stevens-Johnson syndrome and/or pemphigoid
	4. Vitamin A deficiency
	5. Any external injury to the orbital or periorbital area
	6. A history of eye surgery within the past six months (except refractive surgery)
	7. Impaired blinking due to facial palsy
	8. History of punctal plug or punctal occlusion surgery
	9. Pregnancy, lactation, or plans to conceive
	10. Exclusion at the investigator’s discretion

VAS: Visual analog scale; TBUT: Tear film break-up time.

### Randomization and allocation concealment

For randomization and allocation concealment, a set of random numbers will be generated using computerized SAS block-randomization software (SAS® version 9.3, SAS Institute, Inc., Cary, NC, USA). Sealed, opaque assignment envelopes will be used for allocation concealment [16].

The primary data collected will be used to investigate the impact of expectations on the success of acupuncture treatment. Because participant expectations could affect treatment outcomes, each patient will be queried regarding their perception of acupuncture therapy prior to group allocation [17]: 'How much do you expect that the acupuncture treatment will relieve your dry eye symptoms?’ Participants will be asked to select one point on a 10-point Likert scale (0 not useful to 9 very useful).

## Intervention

### Acupuncture plus usual care group

The acupuncture plus usual care group will be treated three times per week for four weeks. Each session of treatment will include the use of 17 acupuncture points (bilateral BL2, GB14, TE23, Ex1, ST1, GB20, LI4, and LI11, and single GV23). These will be localized according to the 'WHO Standard Acupuncture Point Location in the Western Pacific Region’ protocol [18]. Four experts in the field of alternative medicine who have trained for at least seven years and practiced for at least three years will perform these treatments using 0.20 mm × 30 mm disposable acupuncture needles (Dongbang Acupuncture Inc., Chungnam, Republic of Korea). The '*de-qi*’ sensation will be induced by twisting acupuncture, and the needles will be retained for 20 minutes before removal. The patients will be allowed to use any additional treatment for dry eye symptoms other than punctal plugs or punctal occlusion surgery. Patients will not be permitted to participate in any other regimen of acupuncture therapy. Any changes in medical history or vital signs and reports of adverse events will be recorded at every follow-up visit.

### Usual care control group

The control group will be allowed to use any type of treatment for dry eye symptoms: artificial tear drops, drugs, supplements (for example, cyclosporine, corticosteroids, and autologous serum), and alternative treatments other than those listed above as exclusions. Patients will be followed-up every two weeks and asked to report any change in medical history or drug use at each visit.

### Primary outcome

#### Ocular Surface Disease Index (OSDI)

The primary outcome will be scores on the OSDI, which is a questionnaire consisting of 12 questions for evaluating the effects of dry eye syndrome on vision, ocular symptoms, and any condition associated with dry eye. The patient will answer each question on a scale ranging from 0 to 4, with 0 indicating 'none of the time’ and 4 indicating 'all of the time’. If a certain question is deemed irrelevant, it will be marked as 'not applicable (N/A)’ and excluded from the analysis. The OSDI score is calculated according to the following formula [19]:

$$\mathrm{OSDI}=\frac{\left(\text{Sum}\;\text{of}\;\text{scores}\;\text{for}\;\text{all}\;\text{questions}\;\text{answered}\right)\;\times \;100}{\left(\text{Total}\;\text{number}\;\text{of}\;\text{questions}\;\text{answered}\right)\;\times \;4}$$

The scale ranges from 0 to 100, with higher scores representing more severe cases of dry eye syndrome [20]. This value will be checked during visits 2 (prior to acupuncture treatment), 8, 14, and 15.

### Secondary outcomes

#### Visual Analog Scale (VAS)

The 100-mm VAS is an instrument for the self-assessment of ocular discomfort. Patients will perform these measurements daily for one week. Each participant will mark the level of ocular discomfort (for example, ocular itching, foreign body sensation, burning, pain, and dryness). The VAS score is determined by measuring the distance between the left-hand end of the line and the point marked by the subject. This will be checked during visits 1, 2, 8, 14, and 15. The VAS scores as reported at visit 1 will be used for screening purposes. The VAS scores obtained at visit 2 will be used as baseline for the analysis.

#### Quality of Life (QOL)

The QOL scale is used to measure the effect of dry eye on the patient’s quality of life. The question is posed as follows: 'During the last week, how would rate your quality of life, considering any impact of dry eye symptoms?’ The possible responses range from 0 (excellent) to 6 (worst). This will be measured at visits 2, 8, 14, and 15.

#### Tear Film Break-up Time (TBUT)

The TBUT test assesses tear film stability. Sodium fluorescein (2.5%) is applied to both eyes, and the time that elapses until the first appearance of a dry spot or disruption in the tear film is measured. A single optometrist performed all TBUT measurements, without holding the participant’s eyelid. TBUT results ≤ 10 seconds suggest dry eye of at least moderate severity. The TBUT will be assessed at visits 1 (screening) and 15.

#### Schirmer-I test (with anesthesia)

The Schirmer-I test (with anesthesia) is used to measure baseline tear secretion. After the application of local anesthesia (Alcaine® Alcon, Inc., Texas, USA) to arrest reflex tearing, Schirmer test paper (Color Bar™ Schirmer tear test, Eagle Vision, Inc., TN, USA) is placed in the lateral third of the lower eyelid. With closed eyes, the patient keeps this paper in place for five minutes, after which the length of the wet portion is measured. This measurement will be obtained during visits 1 (screening) and 15. If the Schirmer-I test result is ≤ 10 mm/5 minutes, the patient’s dry eye is of moderate severity or worse.

### Corneal surface photography

Digital photos will be used to evaluate the corneal surface as revealed by fluorescein staining. After dropping one drop of fluorescein 0.25% in each eye, participants will be asked to blink five times to drain any excess dye. The stained corneal surface will be photographed using the cobalt blue filter and yellow filter on the slit-lamp biomicroscope. The results will be graded according to the Oxford scheme (from 0 (absent) to 5 (severe)) and checked during visits 1 and 15 [21].

### Efficacy of drug or other treatment

From visit 14 onward, all participants will be reminded that they can use any sort of treatment except for surgery or acupuncture. Patients will be asked to record any changes in medical history or treatment regimen in a diary. This will be collected for review at the last visit (visit 15).

### General assessment

A general assessment will be carried out in which both practitioners and participants will assess the improvement in symptoms related to dry eye syndrome after acupuncture or the conventional treatment regimen. The patient’s condition will be graded as excellent, good, fair, no change, or aggravated (Table 2).

**Table 2:** Evaluation plan

	**Screening**	**Treatment or observation**												**Assessment**	
**Week**		**1**			**2**			**3**			**4**			**5**^**a**^	**13**^**b**^
**Visit**	**1**	**2**	**3**	**4**	**5**	**6**	**7**	**8**	**9**	**10**	**11**	**12**	**13**	**14**	**15**
OSDI		**●**						**●**						**●**	**●**
VAS	**●**	**●**						**●**						**●**	**●**
Schirmer-I test	**●**														**●**
TBUT	**●**														**●**
Corneal surface photography	**●**														**●**
QOL		**●**						**●**						**●**	**●**
Drug or therapeutic modalities															**●**
General assessment														**●**	

^a^: 4 weeks after visit 2 + 3 days; ^b^: 12 weeks after visit 2 + 3 days; **●**: All groups.

OSDI: Ocular surface disease index; VAS: Visual analog scale; TBUT: Tear film break-up time; QOL: quality of life.

### Statistical analysis

Because this is an exploratory pilot study designed to evaluate the feasibility of acupuncture for the treatment of dry eye syndrome after refractive surgery, sample size was not calculated according to the conventional power analysis method. Twenty subjects will be recruited for each group.

The statistical analysis will be conducted using SAS software (version 9.3, SAS Institute Inc., Cary, NC, USA) with the significance level set at 0.05. Efficacy measurements were adjusted by intention to treat analysis (ITT). Missing values will be imputed by the last observation carried forward (LOCF) method. Analysis of covariance (ANCOVA) will be used to test measured outcomes (OSDI, TBUT, Schirmer-I test, QOL and VAS for the self-assessment of ocular discomfort). ANCOVA will be used to determine whether dependent variable values are equivalent across levels of a categorical independent variable by statistically controlling for the effects of other variables known as covariates. The change in scores for each group will serve as the dependent variable, the baseline scores will serve as covariates, and the group will serve as the fixed factor. The Chi-square test or Fisher’s exact test will be used to identify any differences among groups in fluorescein staining of the corneal surface.

Using this correlation analysis, we will evaluate the relationship between patients’ expectations of acupuncture treatment and the relief of dry eye symptoms in the acupuncture plus usual care group.

### Adverse events

Adverse events are unexpected signs, symptoms or diseases encountered during the clinical trial, whether or not they are related to acupuncture treatment. Local, general, and psychological adverse events may be observed. Local symptoms may include continual swelling, redness and itching lasting ≥ three days, peripheral neuritis and hemorrhage, and bruises/pain lasting ≥ two weeks. Systemic symptoms include headaches lasting longer than three days and circulatory problems, including dizziness and palpitations. Psychological problems include hypersensitivity for over three days and anxiety or fear for over 60 hours. All unexpected responses related to acupuncture treatment will be recorded. In order to prevent bias, an expert panel of individuals who are not otherwise connected to this study will evaluate any report of an adverse event. Each report will include a description of any associated symptoms, including the date of onset, the duration, and the degree of severity (Spilker’s criteria [22]: 1 = mild, 2 = moderate and 3 = severe, the patient’s perception of a causal relationship with the treatment (1 = definitely related, 2 = probably related, 3 = possibly related, 4 = probably not related, 5 = definitely not related and 6 = unknown), acupuncture-related action (1 = no change, 2 = temporarily suspend, 3 = suspend), treatment of the adverse event and treatment results. If serious adverse events (SAE) occur, these will be related to the IRB; experimental treatments will be stopped immediately and appropriate treatments will be administered.

## Discussion

Approximately 95% of patients suffer from symptoms of dry eye after refractive surgery. These patients may attempt to alleviate their symptoms using artificial tears, punctal occlusion surgery and/or lifestyle modifications [6,23]. Dry eye symptoms after refractive surgery are most commonly caused by iatrogenic corneal nerve damage, which contributes to a loss of corneal sensation [24]. This decrease in sensitivity can result in tear film instability, reduced tear secretion, a decrease in the number of conjunctival goblet cells and/or an increase in tear osmolality. Furthermore, there is evidence that the use of a corneal flap with a superior hinge can decrease corneal sensitivity, potentially leading to a reduction in the blink rate [23].

Prior studies have shown that acupuncture can modulate the autonomic nervous system and immune system, which in turn can increase lacrimal secretion by stimulating lacrimal gland function [14,25-27]. No previous study has examined the effects of acupuncture in the treatment of dry eye syndrome following refractive surgery. This pilot study will show whether acupuncture is effective in the treatment of postoperative dry eye syndrome [9-14]. The results should allow us to evaluate the feasibility of an extensive clinical study and to predict the number of subjects necessary for sufficient power.

## Trial status

Patient recruitment began in January 2013 and will continue until December 2013.

## Abbreviations

ANCOVA: Analysis of covariance; TBUT: Tear film break-up time; IRB: Institutional review board; ITT: Intention to treat; LASEK: Laser-assisted subepithelial keratomileusis; LASIK: Laser *in situ* keratomileusis; LOCF: Last observation carried forward; NECA: The national evidence-based healthcare collaborating agency; OSDI: Ocular surface disease index; PRK: Photorefractive keratectomy; QOL: Quality of life; SAE: Serious adverse event; VAS: Visual analog scale.

## Competing interests

The authors declare that they have no competing interests.

## Authors’ contributions

THK conceived the idea for the study and led protocol development. HJ, SL, ARK, ML and JHL assisted with study concept, study design, clinical interpretation, and manuscript drafting and finalization. JHL provided overall supervision as the primary mentor and led the team in manuscript preparation. HJ wrote this manuscript and approved the final version for publication. All authors have commented upon drafts of the manuscript and have approved the final version of the manuscript.
